# Extraintestinal Manifestations of *Clostridioides difficile* Infections: An Overview

**DOI:** 10.3390/antibiotics14070670

**Published:** 2025-07-02

**Authors:** Konstantinos Mpakogiannis, Fotios S. Fousekis, Stylianos Elemes, Evangelos Mantellos, Eirini Christaki, Konstantinos H. Katsanos

**Affiliations:** 1 Division of Gastroenterology, Department of Internal Medicine, Faculty of Medicine, School of Health Sciences, University of Ioannina, 45110 Ioannina, Greece; kostismpakogiannis@gmail.com (K.M.); fotisfous@gmail.com (F.S.F.); khkostas@hotmail.com (K.H.K.); 2 Division of Medical Oncology, Papageorgiou Hospital, School of Medicine, Aristotle University of Thessaloniki, 56429 Thessaloniki, Greece; stelemes.md@gmail.com; 3 Division of Urology, Hippokration General Hospital of Athens, 11527 Athens, Greece; vagmantel@gmail.com; 4 Department of Internal Medicine, Faculty of Medicine, School of Health Sciences, University of Ioannina, 45110 Ioannina, Greece

**Keywords:** *Clostridioides difficile*, *Clostridium difficile*, extraintestinal manifestations, extraintestinal infections

## Abstract

Introduction: *Clostridioides difficile* (*C. difficile*) is primarily associated with colonic disease, including pseudomembranous colitis. However, in rare instances, it may cause extraintestinal infectious and non-infectious manifestations, particularly in immunocompromised patients or those with significant underlying conditions. Search Methods: A literature review was performed using PubMed, Embase, and Researchgate databases up to 15 February 2025. The following search strings were used: “extraintestinal manifestations”, “extracolonic manifestations”, “extraintestinal infections”, “extracolonic infections”, “*Clostridium difficile*”, and “*Clostridioides difficile*”. Results: Extraintestinal manifestations of *C. difficile* appear to represent fewer than 1% of all reported infections. The most frequently reported infectious complications include bacteremia and abdominopelvic infections and abscesses, often involving polymicrobial cultures, with the isolation of *C. difficile* alongside microorganisms typically found in the normal intestinal microbiota. Rare non-infectious cases, such as reactive arthritis, have also been described. The underlying pathogenetic mechanism is believed to involve disruption of the intestinal barrier and translocation of bacteria or toxins to sterile sites. Conclusions: Though rare, extraintestinal *C. difficile* manifestations pose important clinical challenges. Better understanding of their mechanisms is essential for early recognition and appropriate management. Further research is warranted to define potential mechanisms and therapeutic approaches.

## 1. Introduction

*Clostridioides difficile* (*C. difficile*) is a Gram-positive, spore-forming obligate anaerobe, belonging to the phylum *Firmicutes* [1]. *C. difficile* was first identified in 1935 by Hall in the intestinal flora of healthy newborns and was initially considered non-pathogenic [2]. It was first named *Bacillus dificcilis* due to its fastidious growth requirements and later reclassified as *C. difficile* [2,3]. Its clinical significance was not recognized until the late 1970s [4]. Primary *C. difficile* infection is mainly associated with previous antimicrobial use that can decrease the population of non-pathogenic anaerobes which typically reside in the gut, thus favoring the growth of *C. difficile* [5,6,7]. It produces two major toxins, the enterotoxin A and the cytotoxin B, which act synergistically, leading to pseudomembranous colitis [8,9]. Approximately 20% of *C. difficile* strains produce a binary toxin (CDT) encoded by the tcdA and tcdB genes, which is thought to enhance toxin A and toxin B toxicity [10]. Toxin A promotes the release of neutrophils and, in parallel with toxin B, acts on the glycosylate GTP-binding proteins of the enterocyte cytoskeleton actin [11,12,13]. In this way, relaxation of the tight junctions of the enterocyte cytoskeleton, increases fluid leakage in the mucosal surface [11]. During endoscopy, pseudomembranes appear as 2–10 mm whitish-yellow plaques throughout the colon; the small bowel is rarely affected [14,15,16]. The most common clinical manifestations of *C. difficile* infection include fever, abdominal pain, and severe watery diarrhea [17]. Extraintestinal manifestations of *C. difficile*, primarily consisting of infections, although rarely observed, are garnering increasing scientific interest [18,19,20]. This review summarizes the most common extraintestinal manifestations of *C. difficile*, as outlined in the existing literature.

## 2. Search Methodology

A literature review was conducted using the PubMed, Embase, and Researchgate databases up to 15 February 2025, aiming to identify relevant publications addressing extraintestinal infections or manifestations of *C. difficile*. The following search strings were used: “extraintestinal manifestations”, “extracolonic manifestations”, “extraintestinal infections”, “extracolonic infections”, “*Clostridium difficile*”, and “*Clostridioides difficile*”. All selected articles were further screened for additional relevant articles through their reference lists. No language restrictions were applied.

## 3. Results

### 3.1. Risk Factors for Extraintestinal Clostridioides difficile Manifestations

The main risk factors for extraintestinal *C. difficile* manifestations, which are similar to those of intestinal *C. difficile* infection, are summarized in Table 1.

Major risk factors according to the existing literature are the prior use of broad-spectrum antibiotics and advanced age (>65 years) [21]. In addition, the use of proton pump inhibitors and hospitalization, particularly in patients with significant comorbidities indicative of immunosuppression, such as cancer, diabetes mellitus, cardiovascular disease, liver disease, chronic kidney disease, or sickle cell disease, significantly increase the risk of developing extraintestinal manifestations [22,23]. Surgical interventions of the large intestine or intestinal diseases like *C. difficile* colitis can damage the mucosal surface of the large intestine, leading to the translocation of *C. difficile*, possibly via the bloodstream to sites outside the gut [18,24]. Interestingly, the occurrence of extraintestinal manifestations in patients without intestinal barrier disruption underscores the need for further exploration of the roles of toxins A and B, as well as the potential presence of other toxigenic strains in asymptomatic carriers [8,25,26].

### 3.2. Extraintestinal Clostridioides difficile Manifestations

The potential infectious and non-infectious extraintestinal manifestations are discussed below.

#### 3.2.1. Bacteremia and Sepsis-like Syndrome

The first case of *C. difficile* bacteremia was published in 1962 [27]. Subsequently, a significant number of articles were published, giving particular importance to the consideration of bacteremia as a major extraintestinal infection [18,27,28,29,30,31,32,33,34,35,36,37,38,39,40,41,42,43,44,45,46,47,48,49,50,51,52,53,54,55,56]. Blood cultures are more often polymicrobial and isolate *C. difficile* along with other microorganisms of the normal intestinal microbiota, in contrast to monomicrobial bacteremia which is rare [37,40,47,49,51,53,54]. A potential mechanism of polymicrobial bacteremia is the direct transfer of gut bacteria into the bloodstream through the disrupted intestinal mucosal barrier due to inflammation or immunosuppression, while extraintestinal infective foci have been described as a potential hypothesis for monomicrobial bacteremia [23,24,57]. Interestingly, a retrospective cohort study determined that *C. difficile* bacteremia was more common in patients with gastrointestinal disruption due to malignancy compared to patients with gastrointestinal disruption from another cause and patients without gastrointestinal disruption [46]. Additionally, an important literature review reported that the majority of patients with *C. difficile* bacteremia (64%) had underlying liver disease or malignancy, with fever and abdominal pain being the most common presenting symptoms [40]. Bacteremia caused by *C. difficile* has been additionally described and in cases of extraintestinal infections [42,44,45,58]. However, in most of these cases the blood cultures are negative [25]. The most recent review on *C. difficile* bacteremia found no ribotype-specific risk but noted a higher likelihood of toxigenic strains that cause bacteremia [43]. Chatilla et al. and Lowenkron et al. reported a key case series on *C. difficile* sepsis-like syndrome, in which three of seven patients died despite negative blood cultures [31,33]. Sepsis-like syndrome may result from neutrophil chemotaxis triggered by toxin A and disruption of the intestinal barrier, allowing toxins and microbes to enter the bloodstream [59,60]. Although García-Lechuz et al. initially reported a 50% mortality rate for *C. difficile* bacteremia, later studies show 20–40% [18,20,36,40,43]. Due to the clinical significance of clostridial bacteremia in general, prompt treatment of *C. difficile* bacteremia is essential, with broad-spectrum antibiotics, particularly vancomycin and metronidazole being the most effective [20,23,40,43,52,61]. In addition to this, Lee et al. pointed out that three of four patients who died had not received vancomycin or metronidazole, highlighting their importance in the management of *C. difficile* bacteremia [36].

#### 3.2.2. Abdominal and Pelvic Infections and Abscesses

Numerous publications have discussed cases of patients with *C. difficile*-related abdominopelvic infections and abscesses [57,58,62,63,64,65,66,67,68,69,70,71,72,73,74,75,76,77,78,79,80,81,82,83,84,85]. Immunosuppression due to liver disease, kidney disease, diabetes mellitus, and hemodialysis, as well as the disruption of the intestinal barrier, are major risk factors for the development of abdominal and pelvic infections and abscesses [85,86,87,88]. Stieglbauer et al. reported a splenic abscess in a patient with atrial fibrillation, suggesting that thromboembolism led to splenic infarction and abscess formation [77]. Splenic abscess may also result from bacteremia and trauma [58,67,76]. Bacteremia can precede the formation of the abscess by months [58]. This is supportive of the finding that although many extraintestinal infections are believed to be caused by hematogenous dissemination of *C. difficile*, blood culture at the time of diagnosis is frequently negative [25]. Diagnosis of splenic abscesses caused by *C. difficile* remains challenging due to non-specific symptoms (fever, weight loss, abdominal pain) and laboratory findings (elevated white blood cell count, elevated or normal CRP value) [65,67,73]. An important study found that patients with *C. difficile*-related splenic abscesses had higher IgG and IgA levels against toxin A than those with only diarrhea, though the clinical relevance of these antibodies remains unclear [77]. Reports of pancreatic and liver abscesses due to *C. difficile* are rare [42,51,66,69,71,73,78]. The first pancreatic abscess case involved a 68-year-old man without prior antibiotics or diarrhea; *C. difficile* was cultured from a drained cyst, and the authors suggested hematogenous spread as the likely mechanism of abscess and pylephlebitis development [78]. Liver abscess may also occur through skin injury or a catheter, or via interventions like cholecystectomy and carotid angioplasty, procedures that can introduce spores of *C. difficile* into circulation [42,66,71,74]. The six patients with liver abscess caused by *C. difficile*, whose cases are reported in the current literature, presented in the hospital with fever and localized abdominal pain, while five of them showed elevated C-reactive protein levels as well as impaired liver function tests [42,51,69,71,74]. The diagnosis was primarily established through CT imaging, followed by biopsy and abscess fluid culture [51,66,69,71,74]. In one case report, diagnosis was made by the use of mass spectrometry [42]. Two of the six patients revealed polymicrobial growth in the abscess fluid culture, possibly associated with the history of previous *C. difficile* colitis [51,69]. Liver abscesses are associated with a mortality rate up to 20%, necessitating mandatory treatment [89,90]. It should be mentioned that cases of abdominal wall abscess [20,64], intra-abdominal abscess [52,57], renal abscess [25], periappendicular abscess [57,62], retroperitoneal abscess [72], perianal abscess [52,57], pelvic abscess [68,70], small-bowel abscess [81], tubo-ovarian abscess [82], have also been described in the literature. Chung et al. also ascertained that the most frequent types of extraintestinal infections in their study were abdominopelvic infections [46]. This finding is in agreement with the studies conducted by Matilla et al. and Gupta et al. [20,57]. Intra-abdominal infections and abscesses are frequently polymicrobial [62]. This is supported by findings from Gupta et al., who reported that 62.5% of isolates from such infections involved more than one microorganism [20]. Polymicrobial infections and abscesses may be associated with a positive history of disruption of the intestinal barrier integrity, which enhances the entry of *C. difficile* alongside microorganisms of intestinal microbiota into the bloodstream [20,52,57,58]. A recent study reported *Escherichia coli* and *Enterococcus faecalis/faecium* as the most frequently co-isolated pathogens in such infections, while all *C. difficile* strains from intra-abdominal infections were toxigenic, with ribotype 027, which is often linked to extraintestinal infections, being the most common, likely due to its hypervirulence [25,91,92]. Management of intra-abdominal infections and abscesses typically involves drainage and antibiotic therapy with agents such as metronidazole and vancomycin, often combined with broad-spectrum antibiotics like piperacillin/tazobactam or meropenem, though surgical intervention may be required in more severe or complicated cases [20,42,46,51,52,57,58,62,65,66,67,69,70,71,72,73,76,80,81,82,84,93].

#### 3.2.3. Extra-Abdominal and Extra-Pelvic Infections and Abscesses

##### Central Nervous System (CNS) Infections

Central nervous system (CNS) involvement by *C. difficile* is exceptionally rare, with only two published cases of brain abscesses identified in our review [18,94]. The first case report involved a patient with a brain abscess secondary to suppurative otitis media, in which *C. difficile*, *Streptococcus milleri*, and *Bacteroides intermedius* were isolated from the abscess capsule and purulent material [18]. The patient was treated with IV ceftriaxone and metronidazole [18]. Another important case report included a patient with intestinal *C. difficile* carriage and previous subdural hematoma [94]. The diagnosis carried out with CT imaging along with the abscess fluid culture revealed monomicrobial growth of *C. difficile* [94]. The patient’s history was negative for concomitant diarrhea [94]. The cytotoxicity assay for the *C. difficile* toxin was negative, suggesting that low intestinal virulence of non-toxigenic strains enables their prolonged intestinal carriage, which can be followed by opportunistic infections [94]. Treatment was finally achieved after intravenous vancomycin and ornidazole administration [94]. These cases highlight the diagnostic challenges of extraintestinal *C. difficile* CNS infections, which may occur in the absence of gastrointestinal symptoms and may involve non-toxigenic strains. Their rarity and potential for atypical presentation necessitate a high index of suspicion, particularly in patients with neurosurgical history or other predisposing factors.

##### Pleuropulmonary Infections (Pleural Effusion/Empyema)

Iatrogenic infection is the most likely cause of pleuropulmonary infections caused by *C. difficile*, as the most reported cases are linked to invasive procedures like hernia surgery, thoracentesis, thoracotomy, or chest injuries [95,96,97,98]. The first two pleural effusion cases involved severe pleuritis and pneumothorax, with the respiratory tract suggested as the primary infection source in these and most later cases [99,100,101,102,103]. Notably, *C. difficile* toxin was negative in stool samples, in most published cases, supporting the hypothesis that in cases of pleural infections, *C. difficile* is introduced through the respiratory tract [99,101,102,103]. Rodriguez et al. reported a lung empyema case in a patient with *C. difficile* colitis but without toxin testing, leaving unclear any link between the empyema and prior colitis [104]. Only two of the published case reports with pleural effusion/empyema caused by *C. difficile* revealed polymicrobial growth in the drained pleural fluid [100,105]. Given the severity of clostridial pleuropulmonary infections, which can progress to necrotizing pneumonia and septic shock, prompt initiation of treatment is essential [97,98]. Treatment for pleuropulmonary *C. difficile* infection includes drainage, chest tube insertion, and antibiotics, preferably vancomycin or metronidazole [95,96,99,100,101,102,103,104,105,106]. Alonso et al. reported a case successfully treated with drainage and doxycycline administration, suggesting alternative promising antibiotic options [99].

##### Oral Cavity Infections

Cases of oral cavity infections, such as peritonsillar abscess and inflammation of the salivary gland, have been reported [25,46]. The salivary gland inflammation case revealed non-toxigenic *C. difficile* in fluid culture, possibly as part of the oral cavity’s complex bacterial microbiota [25].

##### Bone and Joint Infections

Bone and joint infections are frequently observed as extraintestinal manifestations of *C. difficile* infections [20,45,52,107,108,109,110,111,112,113,114,115,116,117,118,119,120,121,122,123,124,125]. The majority of septic arthritis cases (nine cases) primarily affect prosthetic joints [110,111,112,113,114,115,116,117,118] rather than native ones (four cases) [45,107,108,109]. The only three published cases involved native joint septic arthritis, observed in patients with sickle cell disease, all presenting with elevated CRP, leukocytosis, and confirmed by MRI and *C. difficile* isolation from blood or/and joint fluid [45,107,108,109]. In sickle cell patients, septic arthritis may result from bloodstream translocation due to immune dysfunction and intestinal ischemia from microinfarcts [126]. However, bacteremia often precedes septic arthritis and blood cultures remain negative in such cases [109]. Additional risk factors of *C. difficile* septic arthritis are active chemotherapy, previous history of cancer (e.g., osteosarcoma, colon cancer, acute lymphoblastic leukemia), chronic kidney disease, alcoholic hepatitis, AIDS, diabetes mellitus, and trauma [108,110,111,112,113,114,115,116,117,118]. The potential pathogenetic mechanism of prosthetic joint septic arthritis caused by *C. difficile* is the direct inoculation of the bacteria during surgery [110,111,112,113,114,115,116,117,118]. Furthermore, surgical procedures near joints may disrupt local vasculature and cause localized immunosuppression, facilitating *C. difficile* growth [108]. In cases of prosthetic joint septic arthritis, *C. difficile* isolation was ultimately accomplished through drained joint fluid or prosthetic joint tissue culture, as blood cultures are often negative in these cases [110,111,112,113,114,115,116,117,118]. Cultural analysis of prosthetic septic arthritis cases revealed monomicrobial growth, further supporting the hypothesis of direct *C. difficile* inoculation during surgery [110,112,113,114,115,116,117,118], while in one case Staphylococcus aureus was also isolated and was found to be possibly associated with a history of traumatic fracture [111]. Bone infections caused by *C. difficile* are typically monomicrobial as well [119,120,121,122,123,124,125]. In 1982, Riley and Khartigasu reported the first case of chronic osteomyelitis in a 21-year-old man following a femur fracture, possibly due to hospital-acquired *C. difficile* [119]. A second case involved a 77-year-old woman with chronic vertebral osteomyelitis, likely resulting from hematogenous spread due to the vertebrae’s rich blood supply, despite no gastrointestinal symptoms [124]. The same mechanism may explain vertebral osteomyelitis in a 17-year-old girl with a history of *C. difficile* colitis and spinal surgery, though direct nosocomial inoculation is also possible, as *C. difficile* was isolated from her wound [123]. In cases of *C. difficile*-related osteomyelitis, the diagnosis was confirmed by isolating *C. difficile* from bone or tissue cultures, while blood cultures were negative [118,119,120,121,122,123,124,125]. According to the existing literature, septic arthritis and osteomyelitis due to *C. difficile* often present a tendency to become chronic [118,119,120,121]. Comprehensive treatment for *C. difficile*-related bone and joint infections should include surgical interventions like drainage and prosthesis removal in septic arthritis cases, combined with antibiotic therapy, primarily metronidazole and adjunctive vancomycin [45,107,108,109,110,111,112,113,114,115,116,117,118,119,120,121,122,123,124,125].

##### Surgical Site and Soft Tissue Infections

Smith et al. reported two cases of cellulitis caused by *C. difficile* following traumatic injuries, where the organism was isolated alongside other Clostridium species or anaerobic streptococci, suggesting direct inoculation through the wounds as a likely mechanism [27]. Similarly, Urban et al. emphasized the role of direct contamination in wound infections following trauma, particularly in a case where *C. difficile* was isolated along with *Bacillus subtilis*, a soil-dwelling organism, supporting environmental contamination as the source [93]. Wound infections linked to toxin A- and toxin B-producing strains were reported in patients with prior trauma [93,127]. Furthermore, a patient with a femoral abscess and leg necrosis from polyarteritis nodosa had a toxigenic *C. difficile* strain isolated from the abscess and a non-toxigenic strain from feces, suggesting direct contamination of the patient’s wound with *C. difficile* spores from the hospital environment [128]. In another case report, toxigenic *C. difficile* ribotype 078 was isolated from a post-surgical non-healing wound, while ribotype 014 was found in the intestinal tract [129]. The presence of different *C. difficile* strains in the gut has been documented, suggesting the possibility of co-infection or missed detection of one strain in stool samples [128,129,130]. Deptula et al. and Kikkawa et al. identified strains producing only toxin A or only toxin B, respectively, suggesting that intestinal colonization by these variants may have contributed to wound infections during or after surgical procedures [131,132]. The first case of *C. difficile* necrotizing fasciitis included a 59-year-old woman who previously had a motor vehicle accident, suggesting that the source of infection was most likely the environment [26]. Necrotizing peritonitis has been reported as a sequela of *C. difficile* colitis, although the microorganism was not identified in cultures of infected soft tissue samples, with dissemination through the femoral duct being a potential pathogenic mechanism [133,134]. In summary, trauma and hospitalization are key factors in *C. difficile*-related soft tissue infections, with the potential mechanism being direct inoculation into wounds from the accident or hospital environment, and less often from the gastrointestinal tract [26,27,93,127,128,129,130,131,132]. Regarding treatment of soft tissue infection, there is no standardized treatment [26]. Intravenous administration of metronidazole could be the key drug, possibly along with wound/tissue irrigation [129,132]. Nevertheless, given that metronidazole proved ineffective in one case, it is advisable for medication to be guided by antibiotic susceptibility [93,131].

##### Cardiovascular Infections

Previous cases of mycotic aneurysms caused by anaerobic bacteria or *Clostridial* species have been discussed in the literature [135,136,137]. The first reported case of mycotic aneurysm caused by *C. difficile*, positive for both toxins A and B, involved a patient without recent surgery, hospitalization, trauma, or antibiotic use, but with diverticulosis [28,138]. This suggests that the patient may have been an asymptomatic *C. difficile* carrier, possibly acquired from the community, aligning with the view that a small percentage of adults without prior antibiotic exposure may harbor *C. difficile* in their intestinal microbiota [28,138]. Chaudry et al. reported the first case of *C. difficile*-related intracardiac vegetation, highlighting that it could be nosocomially acquired due to the patient’s prior hospitalization [139]. Hematogenous spread and bacteremia originating from the intestinal tract are considered the most likely mechanisms behind *C. difficile*-associated cardiovascular infections, as evidenced by reported cases of mycotic aneurysms, pericarditis, infected aneurysms, endovascular graft infections, epicardial patch infections, and pacemaker-related infections [138,139,140,141,142,143,144,145,146]. In rare instances, *C. difficile* bacteremia may precede the onset of extraintestinal manifestations by a prolonged period, as demonstrated by a case reported by Samsky et al., in which a patient developed a *C. difficile*-infected epicardial patch six months after a documented episode of *C. difficile* bacteremia [145]. The likely origin of *C. difficile* in associated cardiovascular infections was the intestinal tract, particularly in patients with confirmed pseudomembranous enterocolitis, detectable toxin in the stools, or other intestinal diseases, such as ischemic colitis, toxic megacolon, diverticulitis, and ulcer at the anastomotic site of previous bowel resection [138,140,141,142,143,144,145,146]. Previous endovascular interventions such as TEVAR are an additional important risk factor for cardiovascular infections because they can create anaerobic conditions that favor the development of *C. difficile* [142]. The anaerobic conditions that promote vascular C. difficile infections are particularly conducive to atherosclerotic lesions, such as those commonly seen in patients with chronic kidney disease undergoing hemodialysis [140]. Other possible risk factors for cardiovascular infections caused by *C. difficile* that can be considered are hypertension, heart failure, impaired glucose tolerance, alcohol abuse, trauma, and predisposed heart disease [44,138,139,145,146]. The first pacemaker infection case involved a 75-year-old male with prior myocardial infarction, where stool cultures identified two different toxigenic strains and one non-toxigenic strain, while in pacemaker probes and blood cultures only the toxigenic strain was identified [143]. Nevertheless, data are lacking on whether toxigenic strains or some ribotypes translocate more easily [43]. In Berkefeld et al.’s case of pacemaker infection, *C. difficile* may have been acquired via skin colonization from the hospital environment, though bloodborne dissemination of *C. difficile* remains a possibility as Staphylococcus aureus was also isolated [143,147]. The diagnosis in all published cases of cardiovascular infections caused by *C. difficile* was established by monomicrobial growth of *C. difficile* in intraoperative specimen cultures [44,138,139,140,141,142,143,144,145,146]. In only two published cases of cardiovascular infections, blood cultures revealed a positive result [44,143]. The 6-month mortality rate due to mycotic aneurysm caused by *Clostridium septicum* reaches approximately 65–100% without surgical intervention [148]. Therefore, in cases of mycotic aneurysm, infected aneurysm, or infected endovascular graft, caused by *C. difficile*, surgical–vascular intervention is necessary, always along with metronidazole or/and vancomycin administration for at least 6 weeks [44,138,140,141,142,144,149]. Infection of an epicardial patch was treated with patch removal and intravenous vancomycin and metronidazole [145]. The patient with infected intracardial vegetation responded well after intracardiac repair combined with metronidazole administration for 14 days [139,150]. The pacemaker infection was treated after extraction of the pacemaker and antibiotic treatment with vancomycin for a total duration of 7 weeks [143]. Pericarditis was successfully treated after administration of metronidazole for 10 days [146].

### 3.3. Non-Infectious Manifestations

The most commonly reported non-infectious extraintestinal manifestation is reactive arthritis, known as Reiter’s syndrome [151,152,153,154,155,156,157,158,159,160,161,162,163,164,165,166,167,168,169,170,171,172,173,174,175,176,177,178,179,180,181,182,183,184,185,186,187,188,189]. The first case report of reactive arthritis caused by confirmed *C. difficile* colitis was described in 1980, while the most recent case report was published in 2024 [151,187]. Putterman et al. established the following clinical criteria for reactive arthritis diagnosis:

The onset of arthritis in conjunction with or following episodes of diarrhea and/or colitis

- The appearance of diarrhea after systemic antimicrobial therapy usage;
- Confirmed *C. difficile* by either a positive stool culture or toxin assay;
- The exclusion of any other possible diagnosis as well as infectious agent for both arthritis and diarrhea [169].

The primary risk factor for *C. difficile*-associated reactive arthritis is the use of antibiotics, mainly beta-lactams like amoxicillin and ampicillin, combined with beta-lactamase inhibitors [184]. A possible pathogenetic mechanism is the increased permeability of the intestinal wall to bacterial antigens, leading to a systemic inflammatory response and the appearance of arthritis [24]. This pathophysiological mechanism may be enhanced by the cytotoxic action of toxin A and the presence of positive HLA-B27 antigen [190,191,192]. Moreover, recent reviews have highlighted that more than fifty percent of patients with *C. difficile*-associated arthritis tested positive for HLA-B27 antigens [181,184]. However, the role of this specific antigen in the pathogenesis of reactive arthritis is still considered debatable [193]. Inflammatory markers like Interleukin 17 and 23, involved in *C. difficile* immunopathogenesis, may also hold a crucial role in inducing reactive arthritis, potentially serving as future prognostic factors of *C. difficile*-associated reactive arthritis [184,194,195,196]. Promising prognostic markers could also be the presence of toxins and 16S RNA of *C. difficile* in joint fluid and the detection of *C. difficile* S-layer protein (SlpA) in serum, as well as the calprotectin levels in plasma and synovial fluid [176,181,184,197,198,199]. Reactive arthritis from *C. difficile* usually appears 10 days after infection, lasting around 40 days, and typically affects the wrist, ankle, and knee in a polyarticular or oligoarticular pattern [24,184]. Regarding treatment, administration of analgesics or/and non-steroid anti-inflammatory drugs is sufficient, leading to a favorable outcome in almost 90% of patients [181,188]. The current literature indicates that antibiotic therapy does not provide superior benefits, unless there is clear evidence of an active infection, in which case antibiotics should be administered [188,200]. In a small percentage of patients, corticosteroids, disease-modifying antirheumatic drugs (DMARDs), may be required [188]. Rare non-infectious extraintestinal manifestations of *C. difficile*, such as Takotsubo syndrome, leukocytoclastic vasculitis, and SIADH, have been reported, highlighting the need for further research on *C. difficile*-related catecholamine toxicity and microvascular dysfunction [201,202,203,204,205,206,207]. Infectious and non-infectious extraintestinal manifestations are summarized in Table 2, while the potential pathogenetic mechanisms contributing to these manifestations are summarized in Table 3. The major characteristics of extraintestinal manifestations are also outlined in Table 4.

## 4. Conclusions

*Clostridioides difficile*, primarily known for causing pseudomembranous colitis, can also lead to extraintestinal manifestations, though these are rare. The most common extraintestinal complications include infections. These extraintestinal infections usually account for less than 1% of *C. difficile* infections and usually involve bacteremia and abdominopelvic infections, predominantly affecting patients with severe underlying conditions [208]. The primary potential mechanism is thought to involve the translocation of *C. difficile* from the intestinal lumen through a compromised intestinal barrier into the bloodstream, subsequently spreading to extraintestinal sites, where it may be isolated with other microorganisms of the normal intestinal microbiota. However, it is also important to consider the possibility of direct inoculation of *C. difficile* into infectious sites as an alternative route of infection in patients with a history of hospitalization, surgery, or trauma. Given the emergence of non-infectious extraintestinal manifestations, most notably reactive arthritis, the need for a deeper understanding of the known toxins associated with *C. difficile*, as well as the exploration of new toxins and strains, is considered imperative.

## Figures and Tables

**Table 1:** Risk factors for extraintestinal *Clostridioides difficile* manifestations.

Risk Factors for Extraintestinal *Clostridioides difficile* Manifestations
Severe underlying diseases and immunosuppression (e.g., cancer, diabetes mellitus, cardiovascular disease, liver disease, chronic kidney disease, sickle cell disease) Intestinal barrier disruption (e.g., *Clostridium difficile* colitis, gastrointestinal surgery, intestinal perforation) Previous hospitalization Advanced age (>65 years) Use of broad-spectrum antibiotics Proton pump inhibitor use

**Table 2:** Extraintestinal manifestations of *Clostridioides difficile* infections.

Extraintestinal Manifestations
Infectious manifestations Bacteremia and sepsis syndrome Abdominopelvic infections/abscesses (e.g., spleen abscess, liver abscess, pancreatic abscess, abdominal wall abscess, intra-abdominal abscess, renal abscess, periappendicular abscess, perianal abscess, pelvic abscess, small-bowel abscess, tubo-ovarian abscess) Extra-abdominal and extra-pelvic infections/abscesses (brain abscess, oral cavity infections, pleuropulmonary infections/abscess, surgical site and soft tissue infections, bone and joint infections, cardiovascular infections)
Non-Infectious manifestations Reactive arthritis Takotsubo syndrome (limited case reports) Syndrome of inappropriate antidiuretic hormone (SIADH) (single case report) Leukocytoclastic vasculitis (single case report)

**Table 3:** Potential pathogenetic mechanisms of extraintestinal infections and manifestations.

Potential Pathogenetic Mechanisms Involving in Extraintestinal Manifestations	
Disruption of the Intestinal Barrier	—Primary mechanism for most extraintestinal infections —Inflammatory processes like colitis, surgeries, or perforations compromise the mucosal barrier —This allows *C. difficile* bacteria or toxins to translocate into the bloodstream —Particularly important in cases of bacteremia, abdominopelvic infections and abscesses, soft tissue infections, joint infections
Hematogenous Spread	—After entering the bloodstream, *C. difficile* can reach distant organs and tissues —Leads to infections in the liver, spleen, brain, joints, bones, cardiovascular structures (e.g., infected aneurysms, pacemakers)
Direct Inoculation	—Especially in surgical sites or trauma-related wounds, spores from the environment (including hospital surfaces) can be introduced directly into tissues —This mechanism likely accounts for Surgical site infections and soft tissue infections Joint infections Pleuropulmonary infections
Toxin-Mediated Damage	—Toxins A and B increase intestinal permeability and trigger systemic inflammation —In cases like reactive arthritis and possibly Takotsubo syndrome, toxin absorption into the bloodstream may initiate immune-mediated or autonomic responses
Immune-Mediated Reactions	—Reactive arthritis and vasculitis —Systemic immune response to *C. difficile* antigens —HLA-B27 positivity increases susceptibility —Toxins may act as superantigens, promoting autoimmunity

**Table 4:** Major characteristics of extraintestinal *Clostridium difficile* manifestations.

Manifestations	Major Characteristics	Diagnosis	Treatment
Bacteremia and sepsis syndrome	—More common in patients with intestinal barrier disruption due to malignancy patients —20–40% mortality	Blood cultures often polymicrobial, while monomicrobial bacteremia is rare	IV antibiotics (vancomycin, metronidazole)
Intra-abdominal infections/abscesses	—Usually occurs after colitis or other intestinal barrier pathology (e.g., surgery, GI tract perforation) —Abdominopelvic infections are the most frequent type of extraintestinal infections	—Imaging + intra-abdominal (ascitic, pelvic, abscess) fluid culture —>50% of intrabdominal infection cases reveal polymicrobial fluid culture —Blood cultures are usually negative —Most common isolated ribotype of *C. difficile* is the toxigenic ribotype 027 —Most common co-isolates are Enterococcus, *E. coli*	Surgical drainage along with antibiotics (metronidazole, vancomycin, broad-spectrum like piperacillin/tazobactam)
Central nervous system (CNS) infections	—Only two published brain abscess cases —Suspicion in patients with neurosurgical history or other predisposing factors	Abscess fluid culture revealed polymicrobial culture in the one case and monomicrobial culture in the second case	IV ceftriaxone and metronidazole or ornidazole
Pleuropulmonary infections	Mostly iatrogenic, linked to invasive procedures like surgery or chest trauma	Fluid culture is usually monomicrobial	Treatment involves drainage, chest tube, antibiotics (vancomycin, metronidazole), while doxycycline also reported as alternative option
Oral cavity infections	Cases include peritonsillar abscess and salivary gland inflammation	Non-toxigenic strains found, possibly as part of oral microbiota, possibly due to local contamination	Not standardized
Bone and joint infections	—Mostly affects prosthetic joints after direct *C. difficile* inoculation during surgery —Native joint septic arthritis linked to sickle cell disease —Cases of osteomyelitis caused by hematogenous spread or nosocomial inoculation —Septic arthritis and osteomyelitis, often chronic	—Joint fluid or tissue culture reveal monomicrobial culture —Blood cultures are often negative	Treatment includes surgical drainage/prosthesis removal plus antibiotics (metronidazole, vancomycin)
Surgical site and soft tissue infections	—Cellulitis and wound infections after trauma or surgery —Direct inoculation from trauma or contaminated hospital environment suspected	—Both toxigenic and non-toxigenic *C. difficile* strains involved in culture of wound/tissue/abscess fluid, which is usually polymicrobial —Ribotype discordance can be noted between wound and gut strains	Metronidazole IV as main therapeutic option, but antibiotic susceptibility testing advised
Cardiovascular Infections	Usually associated with prior hospitalization, intestinal disease, or vascular intervention	—Intraoperative cultures are usually monomicrobial —Blood cultures are positive in rare cases	Surgical intervention plus prolonged antibiotics (metronidazole and/or vancomycin)
Non-infectious manifestations	—Most common is reactive arthritis (Reiter’s syndrome) following confirmed *C. difficile* colitis —Onset around 10 days post-infection —Typically poly- or oligoarticular, affecting wrist, ankle, knee	Clinical criteria + stool toxin/culture	—NSAIDs/analgesics corticosteroids/DMARDs if needed —Antibiotics if active colitis co-exists
